# Changes in aldehyde dehydrogenase-1 expression during neoadjuvant chemotherapy predict outcome in locally advanced breast cancer

**DOI:** 10.1186/bcr3648

**Published:** 2014-04-24

**Authors:** Muhammad Alamgeer, Vinod Ganju, Beena Kumar, Jane Fox, Stewart Hart, Michelle White, Marion Harris, John Stuckey, Zdenka Prodanovic, Michal Elisabeth Schneider-Kolsky, D Neil Watkins

**Affiliations:** 1 Department of Medical Oncology, Monash Medical Centre, East Bentleigh VIC 3165, Australia; 2 Monash Institute of Medical Research, Monash University, Clayton, Melbourne, VIC 3168, Australia; 3 Department of Pathology, Monash Medical Centre, Clayton, Melbourne, VIC 3168, Australia; 4 Department of Surgery, Monash University, Clayton, Melbourne, VIC 3168, Australia; 5 Monash Health Breast unit, East Bentleigh, Melbourne, VIC 3165, Australia; 6 Department of Diagnostic Imaging, Monash Medical Centre, East Bentleigh, Melbourne, VIC 3165, Australia; 7 Department of Medical Imaging and Radiation Science, Faculty of Medicine, Nursing and Health Sciences, Monash University, Clayton, Melbourne, VIC 3168, Australia

## Abstract

**Introduction:**

Although neoadjuvant chemotherapy (NAC) for locally advanced breast cancer can improve operability and local disease control, there is a lack of reliable biomarkers that predict response to chemotherapy or long-term survival. Since expression of aldehyde dehydrogenase-1 (ALDH1) is associated with the stem-like properties of self-renewal and innate chemoresistance in breast cancer, we asked whether expression in serial tumor samples treated with NAC could identify women more likely to benefit from this therapy.

**Methods:**

Women with locally advanced breast cancer were randomly assigned to receive four cycles of anthracycline-based chemotherapy, followed by four cycles of taxane therapy (Arm A), or the same regimen in reverse order (Arm B). Tumor specimens were collected at baseline, after four cycles, and then at surgical resection. ALDH1 expression was determined by immunohistochemistry and correlated with tumor response using Fisher’s exact test while Kaplan-Meier method was used to calculate survival.

**Results:**

A hundred and nineteen women were enrolled into the study. Fifty seven (48%) were randomized to Arm A and 62 (52%) to Arm B. Most of the women (90%) had ductal carcinoma and 10% had lobular carcinoma. Of these, 26 (22%) achieved a pathological complete response (pCR) after NAC. There was no correlation between baseline ALDH1 expression and tumor grade, stage, hormone receptor, *human epidermal growth factor receptor 2* (*HER2*) status and Ki67 index. ALDH1 negativity at baseline was significantly associated with pCR (*P* = 0.004). The presence of ALDH1(+) cells in the residual tumor cells in non-responding women was strongly predictive of worse overall survival (*P* = 0.024). Moreover, serial analysis of specimens from non-responders showed a marked increase in tumor-specific ALDH1 expression (*P* = 0.028). Overall, there was no survival difference according to the chemotherapy sequence. However, poorly responding tumours from women receiving docetaxel chemotherapy showed an unexpected significant increase in ALDH1 expression.

**Conclusions:**

ALDH1 expression is a useful predictor of chemoresistance. The up-regulation of ALDH1 after NAC predicts poor survival in locally advanced breast cancer. Although the chemotherapy sequence had no effect on overall prognosis, our results suggest that anthracycline-based chemotherapy may be more effective at targeting ALDH1(+) breast cancer cells.

**Trial registration:**

ACTRN12605000588695

## Introduction

The use of neoadjuvant chemotherapy (NAC) in women with locally advanced breast cancer can reduce the tumor size and improve the rates of breast conserving surgery [1,2]. The degree of pathological response to NAC has been shown to correlate with long-term prognosis [3-5], although the precise definition of complete pathological response (pCR) varies across different studies [6-8]. Typically, patients with high grade or triple negative tumors have higher pCR to cytotoxic therapy and, conversely, failure to achieve pCR clearly results in poor long-term outcomes [9,10]. In addition, the utility of markers, such as estrogen receptors (ER) and progesterone receptors (PR) status in the neoadjuvant setting, is not clear, although human epidermal growth factor receptor 2 (*HER2*) gene amplification is associated with better response to neoadjuvant anti-HER2 therapy [11,12]. Given the heterogeneity of breast cancer at a phenotypic and molecular level, and the fact that only 15 to 20% of patients achieve a pCR [13-15], biomarkers that accurately predict a survival benefit from NAC remain a pressing and unmet clinical need.

Aldehyde dehydrogenase-1 (ALDH1) is a cytosolic enzyme, responsible for the metabolism of intracellular aldehydes [16]. Numerous preclinical studies have shown that expression of ALDH1 in tumor cells has been associated with stem-like characteristics, including innate chemoresistance and clonal capacity [17-20]. Moreover, expression of ALDH1 in surgically resected breast cancer is strongly associated with metastasis and poor survival [18,21]. Therefore, expression of ALDH1 may serve as a marker of highly clonogenic, chemoresistant stem-like cells that form the basis for recurrent disease in locally advanced breast cancer.

Since NAC protocols allow for the monitoring of responses to chemotherapy in a carefully controlled manner by analyzing the pathologic response, we investigated whether ALDH1 expression could serve as a useful biomarker in breast cancer treated with chemotherapy. In the current study, we addressed the hypothesis that ALHD1 expression correlates with innate chemoresistance, and that up-regulation of its expression following NAC can predict recurrence and hence survival. We tested our hypothesis by analyzing sequential tumor specimens taken before, during and at the end of NAC in a large cohort of patients treated in a prospective randomized clinical trial. We also explored the prognostic role of different pathological breast cancer subtypes, defined by immunohistochemistry and correlated with ALDH1 expression.

## Methods

### Study design

Women with locally advanced breast cancer (T1-T3, N0-N3, M0) between April 2004 and December 2011 were invited to participate in this study with inclusion criteria of histologically confirmed invasive adenocarcinoma of the breast, age >18, an Eastern Cooperative Oncology Group (ECOG) performance status of 0 or 1, and adequate hematological, renal, hepatic and cardiac function. Subjects were excluded based on a prior history of other neoplasms (except non-melanoma skin cancers), other serious medical conditions, or concurrent participation in any other investigational/experimental drug trial. The study was approved by the human research and ethics committees at all the participating institutions (Monash Medical Centre, Melbourne, Australia and Monash University, Melbourne, Australia) and all participating women provided written and informed consent.

Subjects were randomized to receive either four cycles of FEC100 (fluorouracil 500 mg/m^2^, epirubicin 100 mg/m^2^ and cyclophosphamide 500 mg/m^2^) followed by four cycles of docetaxel (100 mg /m2), (Arm A), or the same therapy in reverse order (docetaxel × 4 followed by FEC100 × 4) (Arm B). Each patient received a total of eight, three-weekly cycles over a period of 24 weeks.

**Figure 1:**
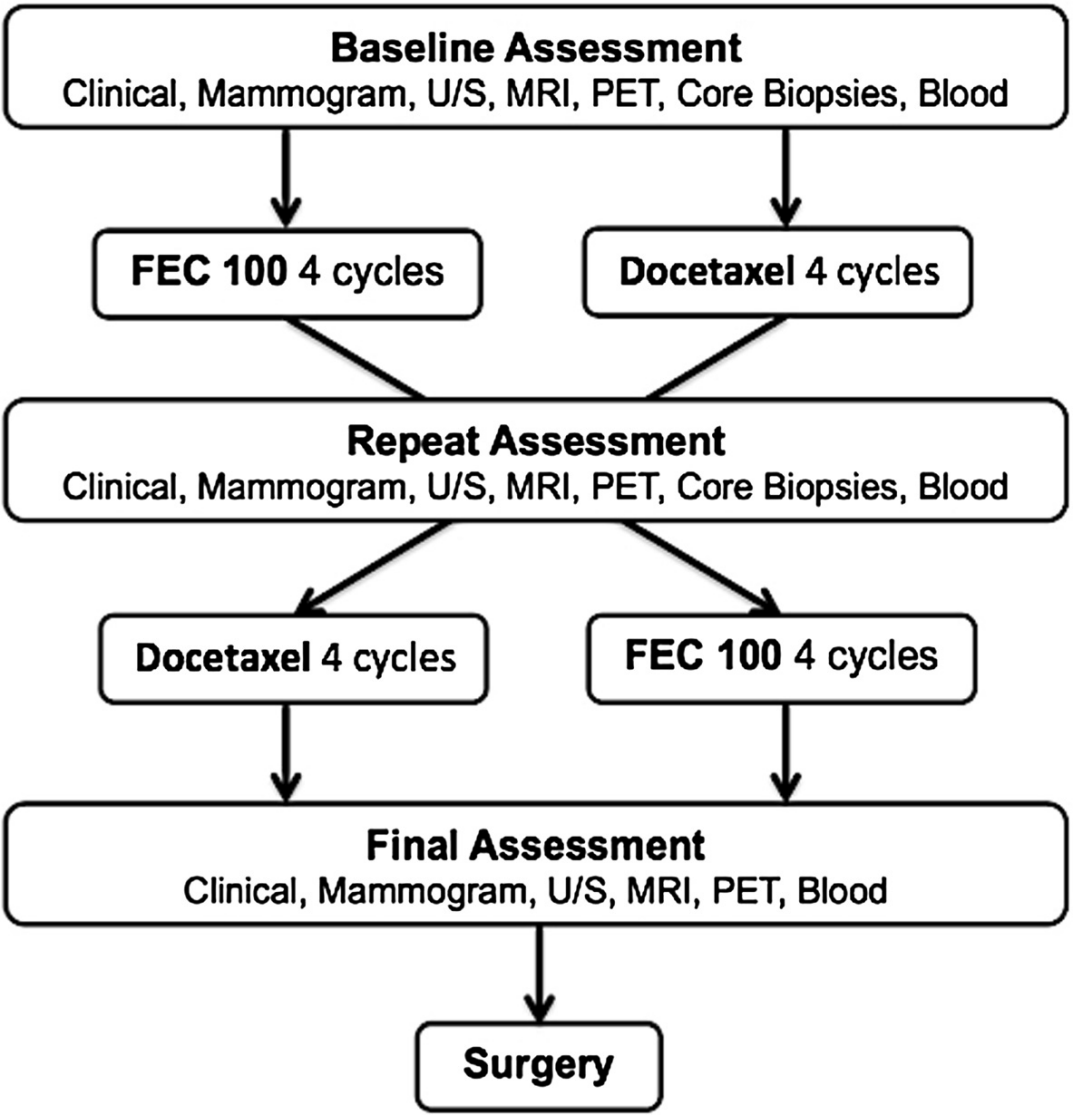
**Study design.** Flowchart depicting the examinations, imaging modalities and chemotherapy for women with locally advanced breast cancer.

All women underwent a clinical examination, mammography, ultrasound and a tumor core biopsy at baseline (within four weeks prior to the commencement of chemotherapy). All assessments were then repeated after four cycles of chemotherapy (FEC100 or docetaxel) and again at the completion of eight cycles (Figure 1). Further periodic clinical assessments after each cycle of chemotherapy were performed to ensure that the tumor was not progressing. All imaging and core biopsies were performed at the same center.

### Specimen collection

Tumor specimens were obtained from each patient at three different time points (Figure 1). The first ultrasound-guided core biopsy was taken at baseline, before any chemotherapy and a second core biopsy was performed after four cycles of chemotherapy. The third sample was obtained from the final surgical excision sample. Each specimen was fixed in 10% formaldehyde and embedded in paraffin. Hematoxylin and eosin (H&E) stained sections were analyzed by an experienced anatomical pathologist, in order to determine tumor type, grade, extent and tissue quality. Each tumor specimen was stained with standard antibodies for the expression of ER, PR, HER2 and Ki67 in accordance with local clinical practice. *HER2* gene amplification was determined by an *in situ* hybridization technique using the INFORM HER2 Dual ISH DNA Probe (Ventana® Medical System, Tucson, AZ, USA). Scoring for *HER2* was performed as per American Society of Clinical Oncology/College of American Pathologists (ASCO/CAP) guidelines [22].

### Breast cancer subtypes

We divided ER^+^ tumors into either luminal A (Her2^−^/Ki67^low^) or luminal B (HER2^+^ or Ki67^high^) subtypes, as previously described [23]. All ER^−^ and HER2^+^ were classified as HER2 subtype, while ER^−^/PR^−^/HER2^−^ was classified as a triple negative subtype [24].

### Immunohistochemistry

The staining for ALDH1 (Vector Laboratories, Burlingame, CA, USA) was performed using Vectastain *Ellite* ABC kit according to the manufacturer’s recommendations. For ALDH1 staining, we used commercially available and previously established monoclonal antibody (clone 44/ALD. BD Biosciences, dilution 1:200) that reliably detects ALDH1 and its isoform ALDH1A1 [25].

All the sections were deparaffinized in xylene and rehydrated in ethanol. Antigen retrieval was performed by microwaving the slides in citrate buffer (pH 6.0) for 10 minutes. Endogenous peroxidase activity was blocked by 15-minute incubation in 1% hydrogen peroxide. A protein block with a 10% normal serum was performed for 30 minutes. Incubation with primary antibody was carried out at 4°C overnight. The secondary antibody was applied for 30 minutes, after washing with tris-buffered saline (TBS). Diaminobezadine solution was used for color detection, followed by counterstaining with hematoxylin. All staining runs were accompanied by appropriate control slides (normal human liver sections).

### Scoring of ALDH1 expression

All the stained slides were scanned into a digital slide scanner (APERIO Scan ScopeXT®, San Diego, CA, USA) and eSlides were created. Magnification of up to 40X was achieved for each section. Two pathologists independently evaluated all the scanned sections in a blinded manner. Whole tumor sections were analyzed thoroughly to look for tumor-specific ALDH1 expression. The ratio of positive to negative cellular profiles was estimated as a percentage of all tumor cells in a slide. The intensity of the staining was also assessed as follows; 0 = no staining, 1 = mild staining, 2 = moderate staining and 3 = strong staining. A histological H-score was obtained by multiplying the percentage of staining with the intensity, thus obtaining an overall score ranging from 0 to 300. In order to classify patients into ALDH1(+) and ALDH1(−) groups, we used the previously published criteria, which was 3+ (≥50% positive tumor cells), 2+ (<50% to ≥10%), 1+ (<10% to ≥5%) and 0 (<5%). For the analysis, all 1+, 2+ and 3+ were considered positive [18,26].

### Pathologic response

A pathological response in the final resected specimens was used as the definitive outcome measure by assessing residual cancer cellularity in all the specimens. A complete pathological response was defined as a complete absence of tumor in the resected primary tumor as well as in the lymph nodes [9].

### Clinical assessment

All tumors and involved axillary nodes were evaluated clinically, by imaging with mammography, ultrasound and fludeoxyglucose-positron emission tomography (FDG-PET) scans at baseline, at the midpoint and at the end of chemotherapy, that is, before surgery. MRI scans were obtained at baseline and at the end of chemotherapy. In this report, only the relevant pathologic data are reviewed.

### Statistical analysis

All analyses were carried out using SPSS, version 21 (SPSS Inc., Chicago, IL, USA). The ALDH1 H-score was compared among patient’s demographic characteristics and baseline tumor characteristics using the Mann–Whitney U test. Sequential changes in ALDH1 H-scores in the corresponding tumor specimens obtained at baseline, at midpoint and at the end of chemotherapy were analyzed using the Kruskal-Wallis test. Patients were also dichotomized into ALDH1(+) and ALDH1(−) groups based on cut-off points as described above. Tumor response rates as well as other clinicopathological parameters were compare among ALDH1(+) and ALDH1(−) groups using Fisher’s exact or Chi square tests, depending on the data characteristics. Overall survival (OS) was defined as the duration (in months) between date of randomization and date of death (due to breast cancer). The Kaplan-Meier method was used to plot the survival curves and the log rank test was used to estimate the statistical difference between the two groups. A Cox proportional hazard model was used to carry out group comparison while the proportional hazard assumption assessment was performed graphically by plotting cumulative hazard functions for the covariates. A *P*-value (two-tailed) of <0.05 was considered statistically significant.

## Results

One hundred and thirty four patients were recruited into the study between April 2004 and December 2011. Of these patients, two withdrew and a further nine were found to have computed tomography (CT) occult distant metastases on FDG-PET imaging, which were confirmed on biopsies. Tumor core biopsies were successfully obtained in 123 patients at the baseline, and from 116 patients at the midpoint of treatment. Baseline core biopsies from four patients contained inadequate tissue for ALDH1 staining and were excluded, leaving a total of 119 informative subjects. Sixty three (53%) patients underwent breast-conserving surgery. All patients were female and most had invasive ductal cancer (108/119, 90%). The majority of the patients had high grade (73/119, 61%) and node-positive (96/119, 81%) tumors. Most of the tumors were positive for estrogen receptors (73/119, 61%) while 30% (36/119) were HER2 positive and 24% (29/119) had a triple negative phenotype. Detailed patient demographics and tumor characteristics of all 119 eligible patients according to the treatment arms are shown in Table 1. All ER positive patients received adjuvant endocrine treatment (tamoxifen or aromatase inhibitors) for at least five years and all HER2 positive patients received trastuzumab for 12 months. All patients received adjuvant radiotherapy to the breast, chest wall and axillary nodes. There was no significant difference in any of the demographic characteristics of the patients in the two chemotherapy arms.

**Table 1:** Patient’s characteristic, overall and according to the treatment arms

**Variable**	**Overall No (%)**	**Arm A *FEC → TAX *** **No (%)**	**Arm B *TAX → FEC *** **No (%)**
Total	119 (100)	62 (52)	57 (48)
Age			
*>50*	67 (56)	22 (36)	30 (53)
*≤50*	52 (44)	40 (64)	27 (47)
Sex			
*Female*	119 (100)	62 (100)	57 (100)
*Male*	0 (0)	0 (0)	0 (0)
T stage			
*T1*	22 (18)	11 (18)	11 (19)
*T2*	78 (65)	41 (66)	37 (64)
*T3*	19 (17)	10 (16)	9 (17)
Nodal status			
*Positive*	96 (81)	51 (82)	45 (78)
*Negative*	23 (19)	11 (18)	12 (22)
Histological type			
*IDC*	108 (88.5)	57 (92)	51 (90)
*ILD*	11 (11.5)	5 (8)	6 (10)
Pathological grade			
*1 or 2*	46 (39)	22 (36)	24 (42)
*3*	73 (61)	40 (65)	33 (58)
Breast cancer subtype			
*Luminal A*	24 (20)	10 (16)	14 (24)
*Luminal B*	49 (41)	25 (40)	24 (42)
*HER2*	17 (14)	12 (19)	5 (9)
*TN*	29 (24)	15 (24)	14 (24)
Ki67 *(N = 106)*			
*>20%*	68 (64)	31 (57)	37 (71)
*≤20%*	38 (36)	23 (43)	15 (29)
Surgery			
*Mastectomy*	56 (47)	27 (44)	29 (51)
*BCS*	63 (53)	35 (56)	28 (49)
Response rates			
*pCR*	26 (22)	14 (23)	12 (21)
*non pCR*	93 (78)	48 (77)	45 (79)

BCS, breast conserving surgery; FEC, fluorouracil, epirubicin, cyclophosphamide; TAX, docetaxel; TN, triple negative.

A complete pathological complete response was observed in 26/119 (22%) of patients, while 93/119 (78%) had residual tumors at the end of chemotherapy. There were 23 relapses (19%) and 19 (16%) breast cancer related deaths in the whole cohort. Univariable and multivariable analyses of response rates according to various prognostic groups are shown in Table 2. As expected, high grade or triple negative tumor type was significantly associated with pCR (*P* = 0.002 and 0.033, respectively). None of the 11 patients with invasive lobular carcinoma (ILC) showed a pCR.

**Table 2:** Univariable and multivariable analysis of response rates according to various prognostic groups (N = 119)

**Variable**	**Univariable analysis**		**Multivariable analysis**	
	**Response rates (%)**	***P*-value**	***P*-value**	**Odds ratio (95% CI)**
Age				
*>50*	15			
*≤50*	27	0.180		
T stage				
*T1*	15			
*T2*	19			
*T3*	39	0.082		
Nodal status				
*Positive*	18			
*Negative*	39	0.046	0.026	4.44 (1.23 to 16.41)
Histological type				
*IDC*	24			
*ILD*	0	0.119		
Pathological grade				
*1 or 2*	2			
*3*	34	<0.0001	0.002	31.25 (3.51 to 250.01)
Breast cancer subtype				
*Luminal A*	8			
*Luminal B*	16			
*HER2*	23			
*TN*	41*(TN vs. others)*	0.008	0.033	3.41 (1.10 to 10.86)
Ki67 *(N = 106)*				
*>20%*	16			
*≤20%*	26	0.236		
Treatment arm				
A	22			
B	21	1.0		
ALDH1				
Positive	10			
Negative	32	0.007	0.004	5.76 (1.76 to 18.45)

ALDH1, aldehyde dehydrogenase-1; HER2, human epidermal growth factor receptor 2; IDC, invasive ductal carcinoma; ILC, invasive lobular carcinoma; TN, triple negative.

### Expression of ALDH1

**Figure 2:**
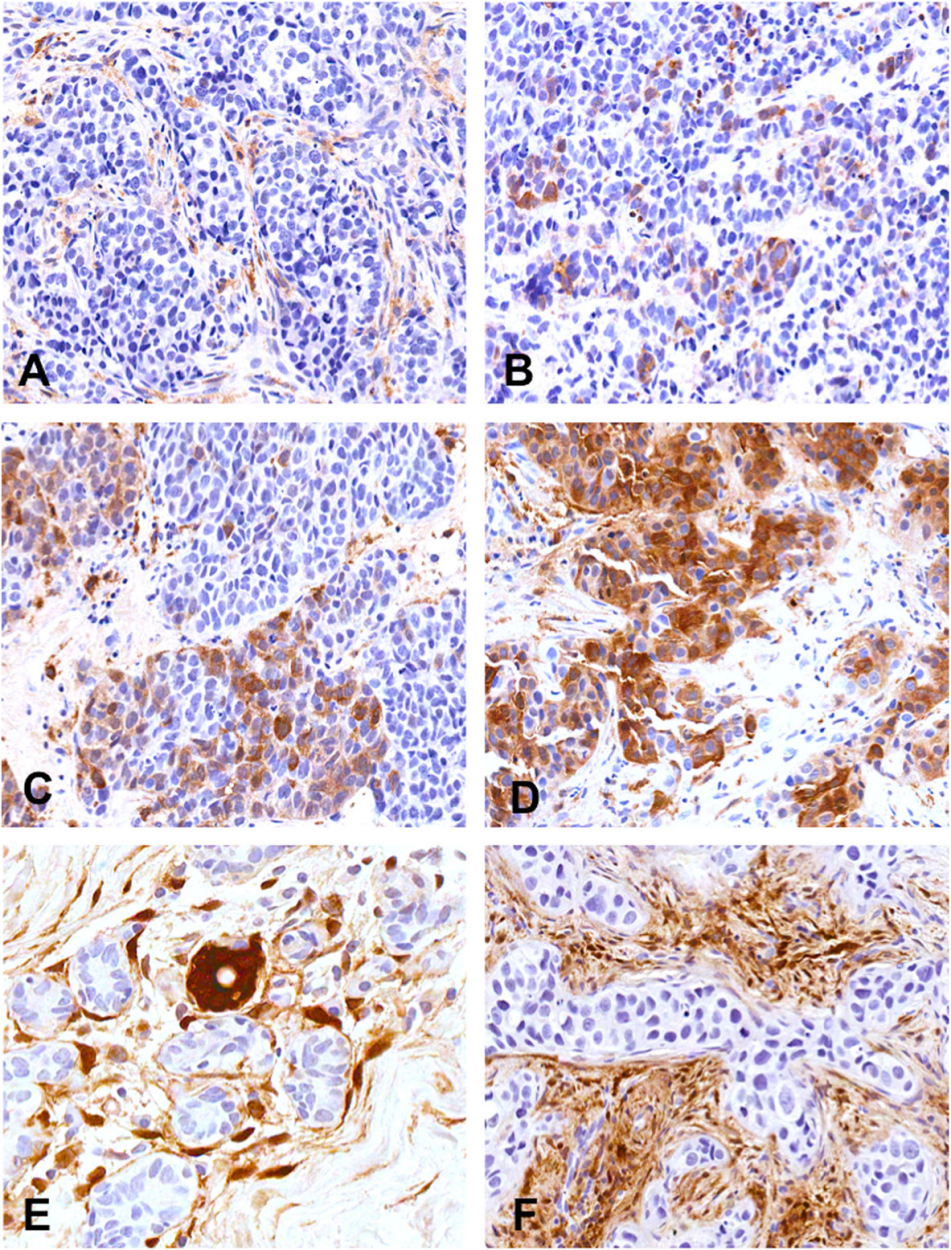
**Representative immunohistochemical staining intensity of ALDH1 for patients with breast cancer. (A)** ALDH1 negative (0) result, **(B)** ALDH1 positive (1+), **(C)** = ALDH1 positive (2+) and **(D)** ALDH1 positive (3+). Strong staining was also observed on the normal ducts **(E)** and tumor stroma in some cases **(F)**. Only cytoplasmic staining in the invasive tumor component was considered for final analysis. ALDH1, aldehyde dehydrogenase-1.

Cytoplasmic ALDH1 staining was observed in the tumor cells, tumor stroma as well as in the areas of *in situ* carcinoma. Strong ALDH1 staining was observed at the bifurcation of the terminal duct lobular unit (TDLU) of the normal ductal epithelium, where stem cells are believed to reside [18]. Tumors staining positive for ADLH1 observed on the invasive cancer component of their tumor were considered positive, while staining in the stroma, areas of ductal carcinoma *in situ* (DCIS) and normal ducts was ignored. At baseline, 56 (47%) patients were ALDH1+; however, only eight patients (7%) showed 3+ (≥50% positive tumor cells) staining. Representative sections are shown in Figure 2.

The correlation of ALDH1 expression at baseline according to various prognostic groups is shown in Table 3. There was no significant association of ALDH1(+) cases with any other parameters, such as patients’ age, tumor stage, tumor grade or breast cancer subtype, though there were relatively higher ALDH1(+) cases in HER2 (59%) and triple negative (55%) subtypes compared to luminal subtypes (41%). Moreover, there was no significant difference in baseline ALDH1 expression when comparing the two chemotherapy arms.

**Table 3:** Correlation of ALDH1 with other prognostic markers

**Variable**	**Total No (%)**	**ALDH1 (+) No (%)**	***P*-value**
Total	119 (100)	56 (47)	
Age			0.100
*>50*	67 (56)	25 (37)	
*≤50*	52 (44)	31 (59)	
T stage			
*T1*	22 (18)	9 (40)	
*T2*	78 (65)	38 (49)	
*T3*	19 (17)	8 (42)	0.985
Nodal status			
*Positive*	96 (81)	46 (50)	
*Negative*	23 (19)	8 (38)	0.455
Histological type			
*IDC*	108 (88.5)	52 (48)	
*ILD*	11 (11.5)	4 (36)	0.537
Pathological grade			
*1 or 2*	46 (39)	22 (48)	
*3*	73 (61)	34 (46)	0.395
Breast cancer subtype			
*Luminal A*	24 (20)	10 (41)	
*Luminal B*	49 (40)	20 (41)	
*HER2*	17 (14)	10 (59)	
*TN*	29 (24)	16 (55)	0.431
Ki67 *(N = 106)*			
*>20%*	68 (64)	29 (58)	
*≤20%*	38 (36)	21 (42)	0.311
Surgery			
*Mastectomy*	56 (47)	21 (37)	
*BCS*	63 (53)	35 (55)	0.066
Response			
*pCR*	26 (22)	6 (23)	
*Non-pCR*	93 (78)	50 (54)	0.007

ALDH1, aldehyde dehydrogenase-1; pCR, pathologic complete response; HER2, human epidermal growth factor receptor 2; IDC, invasive ductal carcinoma; ILC, invasive lobular carcinoma; TN, triple negative.

### Baseline ALDH1 and response to NAC

Low level expression of ALDH1 in baseline biopsy samples strongly correlated with pCR following NAC, with pCR rates of 32% in ALDH1(−) tumors compared to only 10% in ALDH1(+) tumors (*P* = 0.007). In multivariable analysis, negative baseline ALDH1 status was independently associated with complete pathological response to NAC (*P* = 0.004, Odds Ratio 5.76, 95% CI 1.76 to 18.45). These data support similar findings previously reported in breast cancer, suggesting that the expression of ALDH1 is a marker of innate chemoresistance [26]. Patients with high grade tumors or triple negative tumors also achieved significantly higher pCR rates with *P*-values of 0.002 and 0.033, respectively (Table 2).

### Expression of ALDH1 as a prognostic factor in locally advanced breast cancer

**Figure 3:**
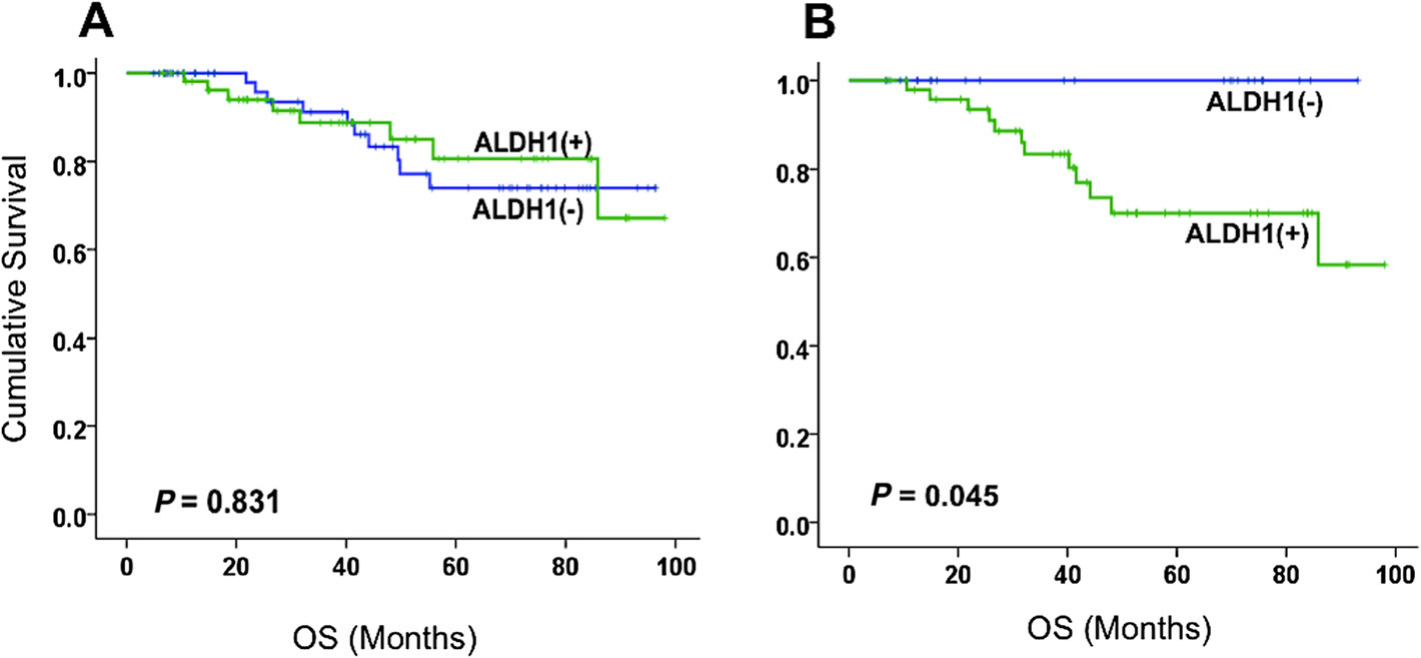
**Overall survival analysis according to ALDH1 expression pre- and post-chemotherapy.** The Kaplan-Meier curves illustrate the prognostic significance of ALDH1 pre and post neo-adjuvant chemotherapy (NAC) in patients with locally advanced breast cancer. **(A)** Before chemotherapy, there was no significant difference in overall survival in ALDH1(−) vs. ALDH1(+) groups. **(B)** At the end of chemotherapy, patients who were ALDH1(−) had much better overall survival (100% five-year survival) compared to those who were ALDH1(+) (66.5% five-year survival). (*P* = 0.045, HR 3.75, 95% CI 1.03 to 14.42). ALDH1, aldehyde dehydrogenase-1.

**Figure 4:**
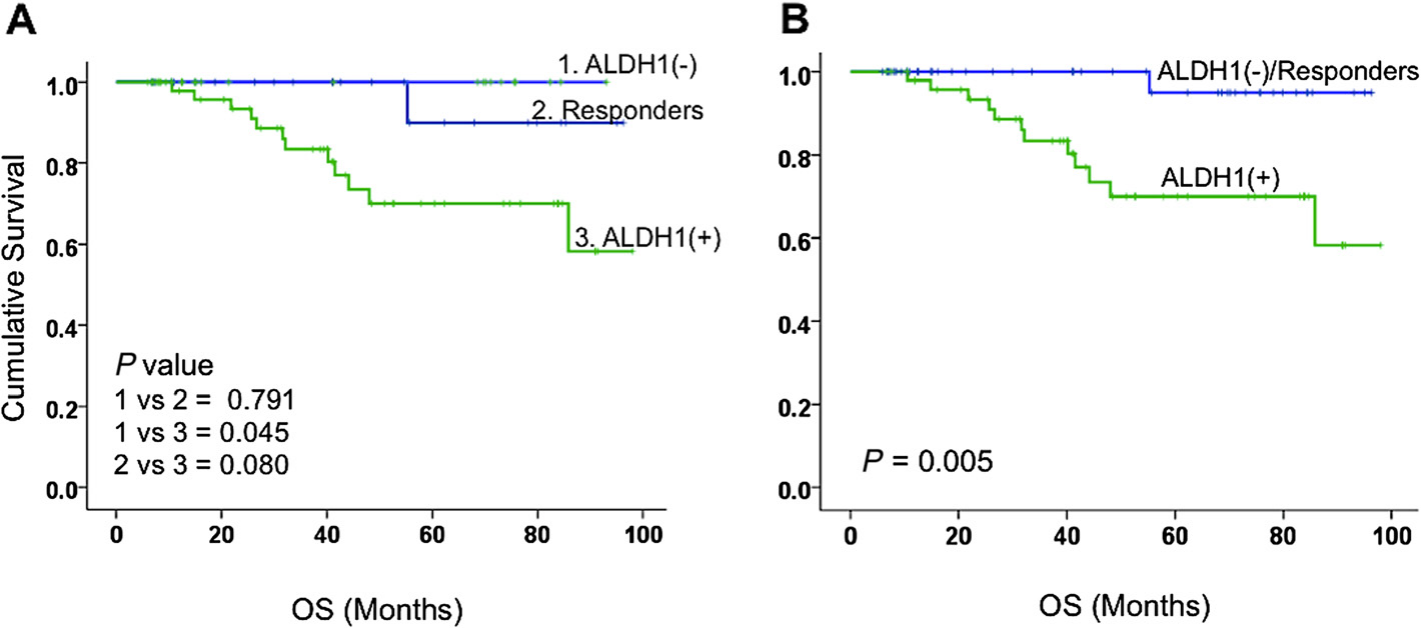
**Prognostic significance of residual tumor at the end of NAC.** These Kaplan-Meier curves depict that at the end of NAC, patients with complete pathological response (pCR) (blue line in **A**) had much better OS compared to those with a residual tumor at the end of NAC (green line in **A**). Surprisingly, patients with ALDH1(−) residual tumors at the end of NAC (purple line in **A**) also had much better OS. When combined into one group (purple line in **B**), patients with no residual tumor or ALDH1(−) residual tumor had significantly better OS compared to those with an ALDH1(+) residual tumor at the end of NAC (*P* = 0.005, HR 10.58, 95% CI 1.65 to 14.68). ALDH1, aldehyde dehydrogenase-1; NAC, neoadjuvant chemotherapy; OS, overall survival.

The prognostic value of ALDH1 was calculated using Kaplan-Meier survival analysis. The results showed that baseline ALDH1 expression was not associated with OS (*P* = 0.831). Patients achieving complete pathological response to NAC showed a non-significant trend towards better OS (*P* = 0.08). Although baseline ALDH1 expression was associated with a response to NAC, it did not predict a long-term prognosis (Figure 3). Interestingly, in women whose tumors were negative for ALDH1 at the conclusion of NAC, OS was remarkably greater than those with residual ALDH1(+) disease (*P* = 0.045) and was almost similar to those achieving a pCR (Figures 3 and 4). In multivariable analysis, neither pCR nor ALDH1(−) residual tumor was a predictor of OS, possibly due to a fewer number of patients. However, when combined into one group, patients with ALDH1(−) tumors at the conclusion of NAC and those achieving pCR, had significantly greater OS compared with those having ALDH1(+) residual disease (*P* = 0.005). In multivariable analysis, this effect was seen independently of patients’ age, tumor stage, tumor grade or chemotherapy sequence (Table 4), and the data suggest that an ALDH1(+) residual tumor at the end of chemotherapy is an independent prognostic factor for survival in locally advanced breast cancer (*P* = 0.024, HR 4.61 95% CI = 1.30 to 23.00).

**Table 4:** Univariable and multivariable analysis of OS according to various prognostic groups

**Variable**	**Univariable analysis**		**Multivariable analysis**	
	**HR (95% CI)**	***P*-value**	**HR (95% CI)**	***P*-value**
**Treatment Arm**				
(A vs. B)	1.56 (0.60 to 4.16)	0.361		
**Age** (>50 vs. ≤50)	1.45 (0.56 to 3.75)	0.441		
**IDC vs. ILC**	1.34 (0.45 to 5.49)	0.462		
**Grade** (3 vs1/2)	1.79 (0.67 to 4.81)	0.244		
**ER** (+) vs. (−)	0.53 (0.21 to 1.35)	0.188		
**PR** (+) vs. (−)	0.31 (0.11 to 0.89)	**0.030**	0.31 (0.10 to 1.19)	0.071
**HER2** (+) vs. (−)	1.22 (0.45 to 3.26)	0.692		
**TN** (yes) vs. (no)	1.75 (0.65 to 4.65)	0.267		
**Ki67** (High) vs. (Low)	1.47 (0.50 to 4.16)	0.480		
**pCR vs. non pCR**	2.21 (0.88 to 6.64)	0.080	1.42 (0.08 to 2.32)	0.801
**ALDH1** (+) vs. (−)				
Baseline	1.10 (0.43 to 2.81)	0.831		
Post NAC*	10.58 (1.65 to 14.68)	**0.005**	4.61 (1.30 to 23.00)	**0.024**

ALDH1, aldehyde dehydrogenase-1; pCR, complete pathologic response; ER, estrogen receptor; HER2, human epidermal growth factor receptor 2; IDC, invasive ductal carcinoma; ILC, invasive lobular carcinoma; PR, progesterone receptor; TN, triple negative. *Post NAC, patients include ALDH1(−) as well as those with pCR.

### Sequential changes in ALDH1 expression following NAC

**Figure 5:**
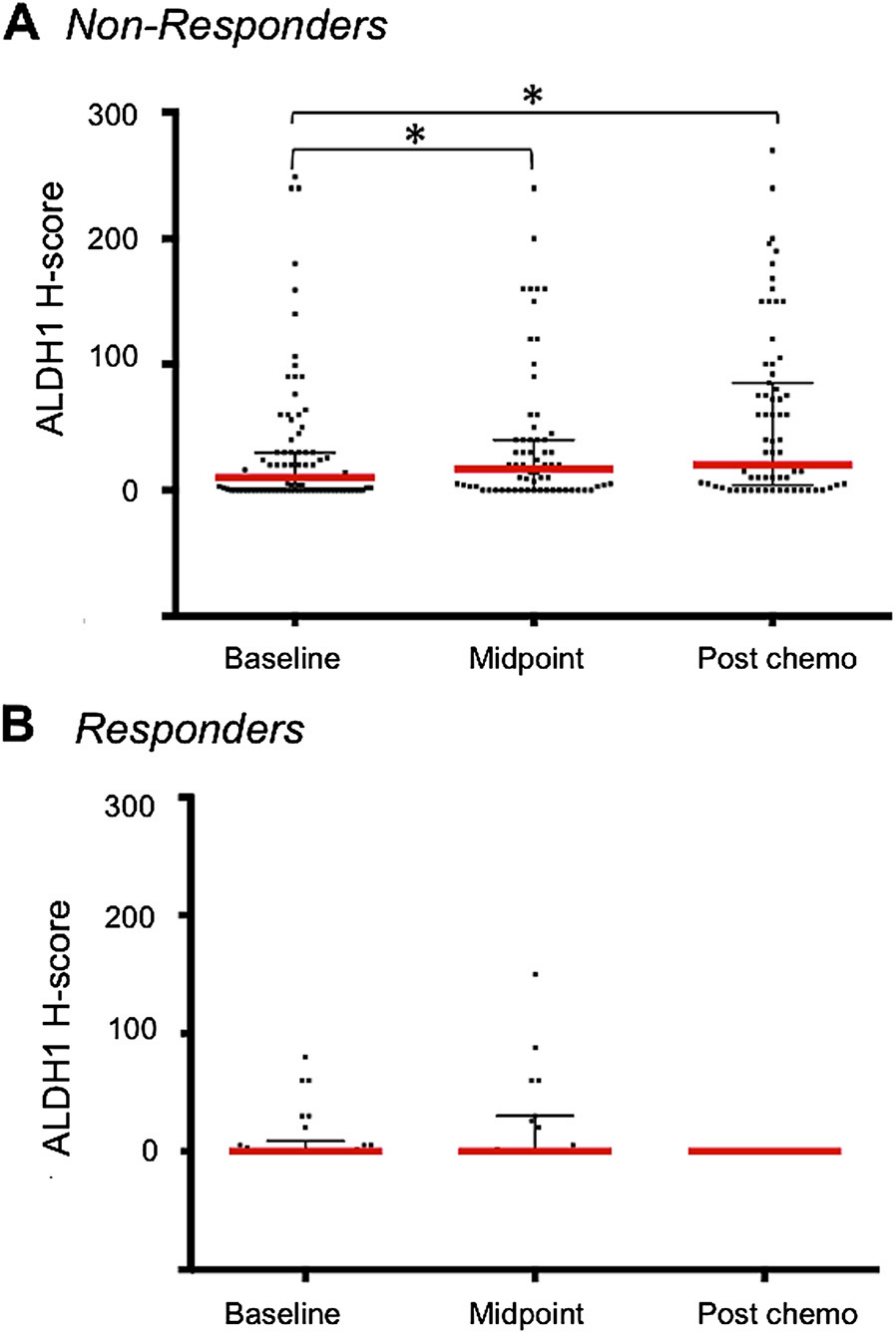
**Illustration of chemoresistance in ALDH1(+) tumors. (A)** Patients who did not have pCR to NAC, there was a statistically significant rise in median ALDH1 H-score at midpoint (after four cycles) as well as at the end (after eight cycles) of NAC. **(B)** Patients who achieved pCR could not be stained for ALDH1 due to lack of tumor cells at the end of eight cycles; however, after four cycles, there was no significant rise in median ALDH1 H-score. Horizontal bars indicate median H score (red) with 10^th^ to 90^th^ percentile (black). (_*_ = *P-*value <0.05, Kruskal Wallis test). ALDH1, aldehyde dehydrogenase-1; pCR, complete pathologic response; NAC, neoadjuvant chemotherapy.

For this analysis, we divided the patients into two groups, those who achieved a pCR such that ALDH1 expression could not be assessed at surgery, and those with residual tumor cells and informative ALDH staining (76 cases). Of those, 50 tumors (66%) were ALDH1(+), with 26% showing strong (3+) staining. In patients who did not achieve a pCR, there was a significant rise in ALDH1 expression following NAC chemotherapy compared to baseline (*P* = 0.028, Kruskal Wallis test) (Figure 5).

### Sequential changes in ALDH1 and the effect on response rates

**Figure 6:**
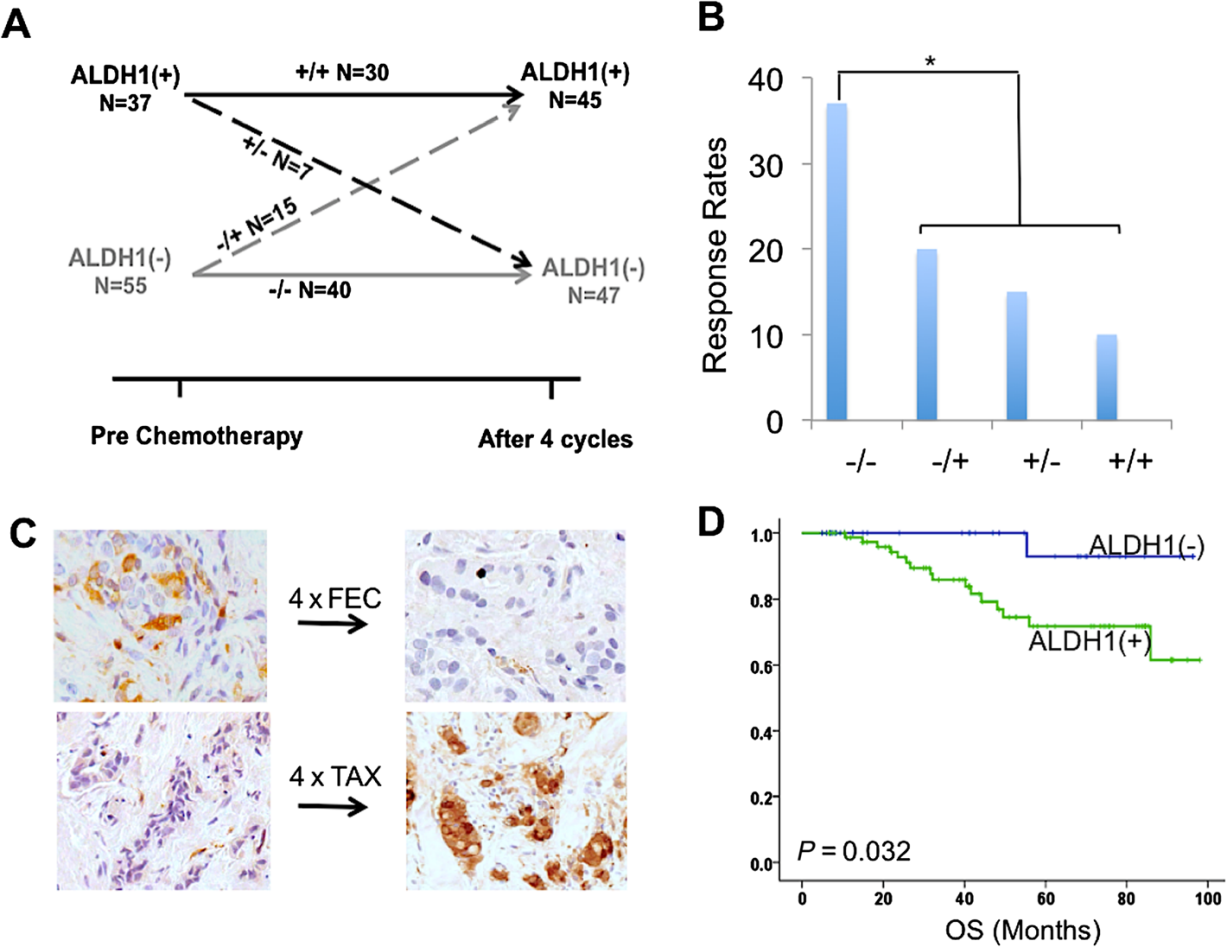
**Illustration of phenotypic switching in response to chemotherapy.** Dynamic changes in the expression of ALDH1 were observed during NAC. Chart **A** illustrates that there was a switch from ALDH1(−) to ALDH1(+) phenotype (positive switch) in 27% of cases and from ALDH1(+) to ALDH1(−) phenotype (negative switch) in 19% of cases, after four cycles of chemotherapy. However, patients who remained ALDH1(−) both at baseline and after four cycles had the highest response rates, while those who were ALDH1(+) at both time points had the lowest response rates. Those who were ALDH1(+) at any time point had intermediate response rates **(B)**. We observed a positive switch more often on patients receiving docetaxel (TAX) and a negative switch more often in patients receiving FEC chemotherapy **(C)**. Finally, the overall survival of patients who remained ALDH1(−) throughout the eight cycles of NAC was significantly higher than those who were ALDH1(+) at any time point **(D)**. (_*_ = *P* <0.05 Fishers exact test). ALDH1, aldehyde dehydrogenase-1; FEC, fluorouracil, epirubicin, cyclophosphamide; NAC, neoadjuvant chemotherapy.

Since we were unable to assess tumor ALDH1 expression in patients achieving a pCR, we next analyzed the core samples obtained at the study midpoint following four cycles of either FEC or docetaxel. Of the 116 patients who underwent tumor core biopsy at midpoint, 24 (21%) had no residual tumors. The remaining 92 (79%) patient samples were stained for ALDH1, 45 (49%) were ALDH1(+). We also observed phenotypic switching in some cases in response to chemotherapy (Figure 6A,C). Of 55 ALDH1(−) cases, some (N = 15, 27%) became ALDH1(+), while the majority (N = 40, 73%) remained ALDH1(−). Similarly, in the ALDH1(+) group (N = 37), the majority (N = 30, 81%) remained ALDH1(+) while seven patients (19%) became ALDH1(−). When combined into one group, patients with ALDH1(+) tumors, either at baseline or at midpoint, had pCR rates that were much worse compared to those who remained ALDH1(−) at both points (37% vs. 16%, *P* = 0.019) (Figure 6B). Remarkably, patients whose tumors remained negative at baseline, midpoint and following completion of NAC had significantly better outcomes despite failing to achieve a pCR (Figure 6D).

### Effect of chemotherapy type on ALDH1 expression and tumor response

**Figure 7:**
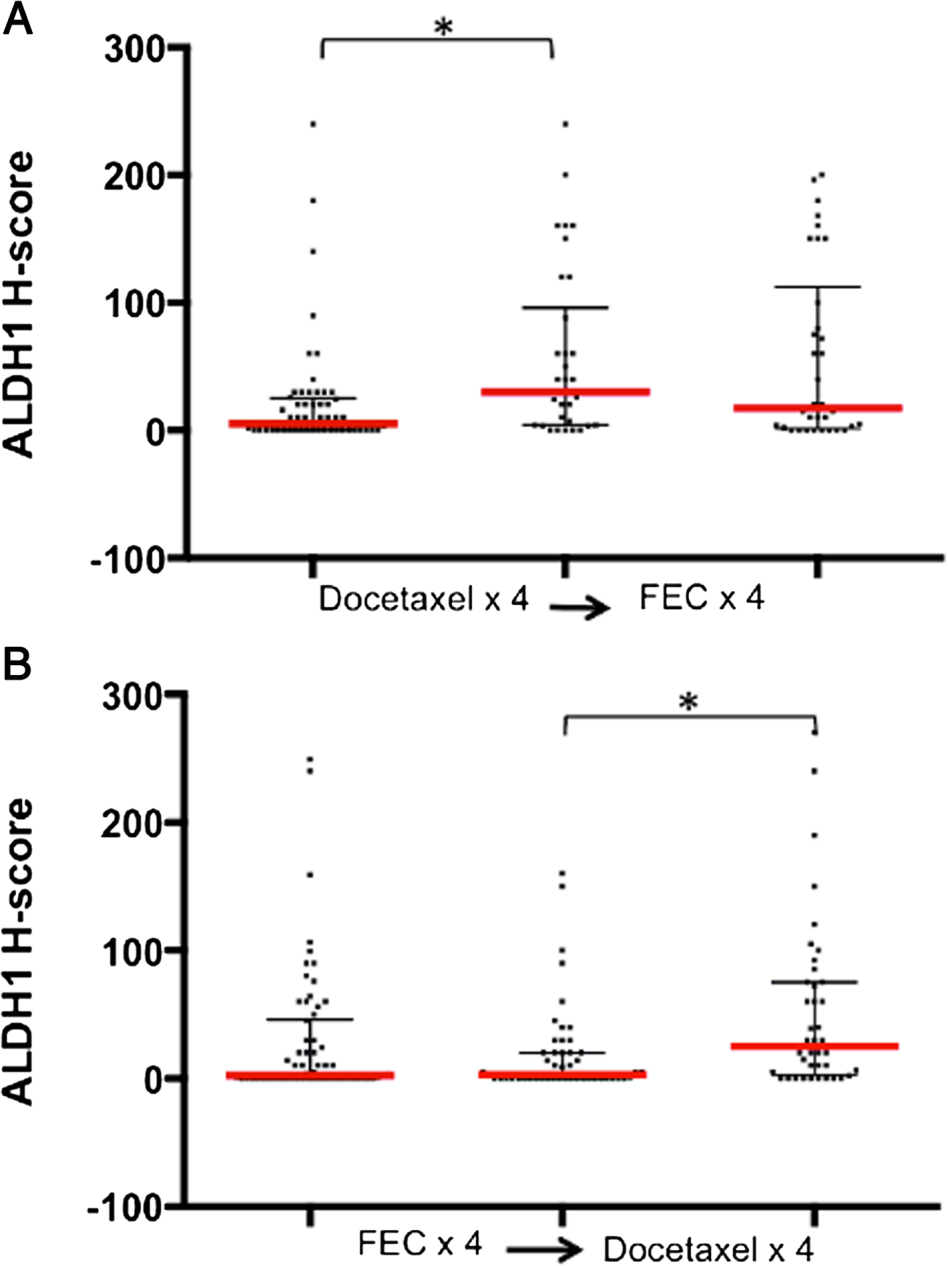
**The effect of chemotherapy type on ALDH1 expression.** There was a significant rise in median ALDH1 H-score after docetaxel (TAX) therapy, whether received at the beginning **(A)** or after four cycles of FEC **(B)**. Conversely, there was no significant change in the median H-score after FEC, whether received at the beginning or after four cycles of docetaxel (TAX). Horizontal bars indicate median H score (red) with 10^th^ to 90^th^ percentile (black). (_*_ = *P*-value <0.05, Mann–Whitney U test). ALDH1, aldehyde dehydrogenase-1; FEC, fluorouracil, epirubicin, cyclophosphamide.

To observe the chemotherapy effect on ALDH1 expression we again focused on the changes in ALDH1 expression between baseline and midpoint samples, and compared the effects of FEC vs. docetaxel on the expression of ALDH1. Unexpectedly, we observed a significant increase in the median ALDH1 H-score in patients who received docetaxel first (*P* = 0.029, Mann Whitney U test) (Figure 7), whereas tumor samples from patients who had received FEC showed no significant difference between ALDH1 expressions. A similar effect was observed in samples from patients who did not achieve a pCR following the full cycles of treatment. Here, tumors from subjects treated with docetaxel in the last four cycles displayed a significant increase in the median ALDH1 score in the final specimen compared to at the midpoint (*P* = 0.002). Once again, this effect was not seen in tumors treated with FEC chemotherapy following docetaxel (*P* = 0.308). Moreover, when we explored the phenotypic switching according to the chemotherapy type, we observed that the switching from ALDH1(−) to ALDH1(+) phenotype was more often seen in tumor samples after four cycles of docetaxel (23%) compared to those receiving four cycles of FEC (10%). The opposite phenomenon was observed more often in the FEC group (20%) compared to the docetaxel group (10%) (*P* = 0.040, Fisher’s exact test).

## Discussion

In a large cohort of women with locally advanced breast cancer treated with NAC, we have shown that expression of ALDH1, a marker of stem-like cancer cells, strongly predicts for innate resistance to sequential FEC/TAX chemotherapy. These findings are in keeping with those of Tanei *et al*., who reported that in 108 patients with locally advanced breast cancer, treated with taxane/anthracyline-based NAC, ALDH1(+) tumors were significantly associated with low pCR rates [26]. This finding also supports the conclusions of preclinical studies showing that ALDH1 expression and/or activity marks breast cancer cells with an innate ability to evade chemotherapy, and a markedly enhanced capacity for tumor regeneration *in vitro* and *in vivo*[18,27].

Surprisingly, we showed that while ALDH1 expression in baseline tumor samples is associated with primary resistance to NAC, it does not predict long-term survival. However, we showed that women in whom residual tumor cells remained, ALDH1(−) derived a major survival benefit. Moreover, when the group of women who achieved a pCR was combined with those with ALDH1(−) tumors at the end of NAC, this group also derived a highly significant survival advantage. Previous similar studies were limited by a retrospective study design and hence variation in results and difficulty in comparing the study outcomes [28,29]. To our knowledge, this is the first time that a biomarker of OS in residual tumor cells that predicts OS in NAC patients has been described in a prospective randomized study. A comparison of major studies [26-30] in locally advanced breast cancer investigating the predictive and prognostic significance of ALDH1 is summarized in Table 5.

**Table 5:** Summary of major studies investigating the clinical significance of ALDH1 in locally advance breast cancer

**Study**	**Chemotherapy**	**No**	**Antibody**	**ALDH1 cutoff**	**% positive**	**Main results**
*Tanie et al. 2009*[26]	Paclitaxel → FEC	108	BD	≥5%	19	• ALDH1(+) but not CD44+/CD24- phenotype is associated with chemoresistance
*Gong et al. 2010*[27]	FEC	192	Abcam	≥20%	19.8	• ALDH1 at baseline correlated with clinical response (CR/PR) and OS
*Sakakibara et al. 2011*[28]	AC → paclitaxel	115	BD	≥5%	39	• ALDH1(+) cells in residual axillary nodes is associated with poor prognosis
*Lee et al. 2011*[29]	AD or AC	92	BD	≥5%	13	• ALDH1(+) but not ALDH1(−) cases had high pCR
						• Increase in ALDH1 after NAC is associated with poor DFS
*Resetkova et al. 2009*[30]	Anthracycline/paclitaxel	34	BD	Any	56	• Stromal but not the tumor expression of ALDH1 is prognostic in breast cancer.
Current study	FEC → TAX Or TAX → FEC	119	BD	≥5%	47	• Pre NAC, ALDH1(+) is associated with poor pCR rates
						• Post NAC, ALDH1(+) cells in residual primary tumor is prognostic
						• Degree of chemoresistance may be different for different chemotherapy types

There was consistency among most of the studies in terms of the primary antibody used and cut-off point utilization. Variation in results could be explained by the differences in study designs and/or patients’ population.

AC, adriamycin (doxorubicin), cyclophosphamide; DFS, disease free survival; FEC, fluorouracil, epirubicin, cyclophosphamide; NAC, neoadjuvant chemotherapy; OS, overall survival; TAX, docetaxel.

Our results show that although patients with ALDH1(+) tumors at baseline display higher chemoresistance than those with ALDH1(−) tumors, ALDH1(−) cases can still convert to ALDH1(+) after chemotherapy and impact the long-term prognosis. This phenomenon can be explained by the likely possibility of the existence of a tiny population (<5%) of ADLH1(+) cells which would have been scored as ALDH1(−) in the baseline samples. Because ALDH1(+) cells are chemoresistant, they could expand at the expanse of their relatively chemosensitive counterparts. Another possible mechanism that can sustain and hence propagate the ALDH1(+) stem cell pool is believed to be the autocrine production of inflammatory cytokines, such as interleukin 6 (IL-6) and interleukin 8 (IL-8), secondary to chemotherapy induced cellular apoptosis [31,32]. The role of IL-6 and IL-8 in the self-renewal of breast cancer stem cells has been extensively studied [33]. Through the activation of STAT-3 and in turn nuclear factor kappa B (NF-κB) signaling in inflammatory cells, these cytokines generate a positive feedback loop between tumor stem cells and immune cells, thus promoting cancer stem cells self-renewal and tumor growth [34]. Interestingly, some initially ALDH1(+) cases also converted to ALDH1(−) after chemotherapy and, hence, achieved improvement in their long-term outcome. These results may support a rare phenomenon of ‘phenotypic switching’, which indicates that stem cell-like and non-stem cell-like populations in breast cancer may be plastic and interconvertible. This phenomenon of cellular plasticity has previously been described in preclinical models [35]. However, this is the first description of the clinical impact of dynamic conversion between marker-defined stem cell-like and non-stem cell-like subpopulations in breast cancer.

This study also demonstrated for the first time that different chemotherapeutic drugs could affect the expression of ALDH1 and subsequent changes in sequential tumor specimens. While there was no difference in the baseline ALDH1 expression in the two groups, patients who received docetaxel and did not respond to treatment, showed more enrichment for ADLH1 expression compared to those who received FEC. Similarly, a significantly higher number of patients switched from ALDH1(−) to ALDH1(+) phenotype post-docetaxel compared to post-FEC. These results suggest that a subgroup of ALDH1(+) patients may receive more benefit from continuing anthracyline-based therapy. However, a further larger study may be needed to prove this hypothesis.

## Conclusions

Our results indicate that ALDH1 is a marker of chemoresistance in locally advanced breast cancer. Enrichment of ALDH1 expression after chemotherapy in non-responding patients suggests self-renewal potential of cancer stem-like cells. However, dynamic changes in ALDH1 expression in response to chemotherapy are a more accurate predictor of long-term survival. Patients remaining ALDH1(+) at the end of chemotherapy have much worse prognosis. The dynamic changes in ALDH1 expression mirror the phenomenon of cellular plasticity where phenotypic switching from ALDH1(+) to ALDH1(−) phenotype or vice versa may depend on the type of chemotherapy used.

## Abbreviations

ALDH1: Aldehyde dehydrogenase 1; ASCO: American Society of Clinical Oncology; CAP: College of American Pathologists; pCR: Pathologic complete response; DNA: Deoxyribonucleic acid; ER: Estrogen receptor; FEC: Fluorouracil, epirubicin, cyclophosphamide; H&E: Hematoxylin and eosin; HER2: Human epidermal growth factor receptor 2; IDC: Invasive ductal carcinoma; ILC: Invasive lobular carcinoma; ISH: *In situ* hybridization; NAC: Neoadjuvant chemotherapy; OS: Overall survival; PR: Progesterone receptor; TAX: Taxotere; TDLU: Terminal duct lobular unit.

## Competing interests

The authors declare that they have no competing interests.

## Authors’ contributions

MA carried out immunohistochemistry, collated all the data and wrote the manuscript. VG, JF, SH, MW and MH designed the study, collected clinical data and revised the manuscript. BK was involved in study design, reviewed and scored the slides and revised the manuscript. JS performed all the biopsies, acquired the imaging data and revised the manuscript. ZP acquired and managed data on e-slides and revised the manuscript. MES analyzed all the data and revised the manuscript. DNW supervised the study, was involved in study design, scored slides and co-wrote the manuscript. All authors have approved the final manuscript for publication and agreed to be accountable for all aspects of the work.
